# Analysis of *Transforming Growth Factor- β1* Expression in Resorptive Lacunae following Orthodontic Tooth Movement in An Animal Model

**DOI:** 10.22074/cellj.2016.4218

**Published:** 2017-02-22

**Authors:** Massoud Seifi, Bahram Kazemi, Sattar Kabiri, Mohammadreza Badiee

**Affiliations:** 1 Marquette University School of Dentistry, Milwaukee, Wisconsin, USA; 2 Cellular and Molecular Research Center, Shahid Beheshti University of Medical Sciences, Tehran, Iran; 3 Department of Orthodontic, Dental School, Shahid Beheshti University of Medical Sciences, Tehran, Iran; 4 Dentofacial Deformities Research Center, Research Institution of Dental Sciences, Shahid Beheshti University of Medical Sciences, Tehran, Iran

**Keywords:** *Transforming growth factor-β1*, Root Resorption, Tooth Movement, Gene expression, Rats

## Abstract

**Objective:**

Root resorption is a complication of orthodontic treatment and till date, there is a dearth of information regarding this issue. The aim of this study was to determine whether the expression of transforming growth factor-β1 (TGF-β1, an inflammatory cytokine) is related to orthodontic force. Moreover, if associated, the expression level may be helpful in differential diagnosis, control and ultimate treatment of the disease.

**Materials and Methods:**

In this experimental study, a total of 24 eight-week-old male Wistar rats were selected randomly. On day 0, an orthodontic appliance, which consisted of a closed coil spring, was ligated to the upper right first molar and incisor. The upper left first molar in these animals was not placed under orthodontic force, thus serving as the control group. On day 21, after anesthesia, the animals were sacrificed. The rats were then divided into two equal groups where the first group was subjected to histological evaluation and the second group to reverse transcriptase-polymerase chain reaction (RT-PCR). Orthodontic tooth movement was measured in both groups to determine the influence of the applied force.

**Results:**

Statistical analysis of data showed a significant root resorption between the experimental group and control group (P<0.05), however, there was no significant difference in the expression level of the inflammatory cytokine, *TGF-β1*.

**Conclusion:**

Based on the findings of this study, we suggest that there is a direct relationship between orthodontic force and orthodontic induced inflammatory root resorption. In addition, no relationship is likely to exist between root resorption and *TGF-β1* expression in the resorptive lacunae.

## Introduction

Orthodontic induced inflammatory root resorption is a partial loss of root structure and has been shown to be a common risk in orthodontic treatment (1,3). Root resorption is a multi-factorial phenomenon that results from a combination of biological effects, genetic predisposition and mechanical factors (1,4,5). Although histological studies have reported a root resorption incidence of 90% in orthodontic treated patients, the severity of root resorption is often clinically lower than this ratio. Moreover, some studies have shown that 1-5% of orthodontic treated teeth develop severe root resorption exceeding 4 mm (1). Loss of root structure can result in insufficient crown-root ratio, which may influence tooth prognosis. Therefore, proper diagnosis and patient selection are essential in reducing these complications. 

Root and bone have structural similarities with root resorption being an inflammatory phenomenon similar to bone resorption, however, root resorption occurs on the root surface (6). An ongoing research in orthodontics is the detection of relationships between inflammatory mediators and root resorption. The exact process of orthodontic induced inflammatory root resorption (OIIRR) is yet to be fully understood. Nevertheless, according to some studies, pro-inflammatory cytokines may play important roles in this process (7). One key cytokine that participates in the inflammatory process is transforming growth factor-β1 (TGF-β1). 

*TGF-β1* is a multifunctional cytokine that can affect bone metabolism. This mediator regulates bone remodeling by modulating osteoblasts and osteoclasts. *TGF-β1* elicits different effects on cellular activities. For instance, it stimulates opposed actions (facilitative and suppressive) on osteoclasts. However, when absent in the bone marrow environment, monocytes do not differentiate into osteoclasts. After bone resorption initiation, *TGF-β1* released from the bone matrix can inhibit the production of osteoclast differentiation factors by osteoblast cells (8). 

We therefore aimed to evaluate the relationship between OIIRR and expression of *TGF-β1* using reverse transcription-polymerase chain reaction (RT-PCR) in rats. 

## Materials and Methods

This experimental study consisted of 24 8-week-old male Wistar rats with an average body weight of 230 g (SD=10), which were randomly assigned to two equal groups of which the first group was used for RT- PCR and the second group was subjected to histological examination. All the 24 rats received the orthodontic force procedure. Since the animals were kept in the same place during the experimental period, no acclimatization period was needed. To assess the effects of the experiment on the whole body, the rats were weighed before and after examination. This study was approved by the Institutional Review Board (IRB) of the Dental School of Shahid Beheshti University of Medical Sciences (No. 1388123-67). 

A split-mouth design was used with the contra lateral side of the mouth serving as the internal control for each specimen. On the first day, the animals were anesthetized by 50 mg/kg Ketamine and 2 mg/kg Xylazine intraperitoneally. The method for orthodontic tooth movement was a modified version of that used by King and Fischlschweiger (9). 

The maxillary right first molars were used as the experimental group and their counterparts on the left side were used as the internal control group. The lateral and palatal surfaces of the maxillary right first molars and both upper incisors were etched with phosphoric acid gel (37.4%) for 30 seconds, wiped with disposable brushes soaked in distilled water and dried with air syringe. 

**Fig.1:**
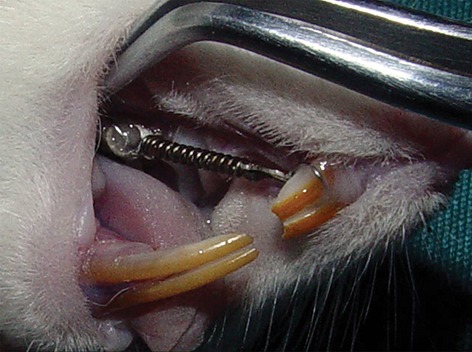
Closed Nickel-Titanium coil spring between first right molar and incisors.

A ligature wire was tied around the cervical third of the first molar and tightened to the closed Nickel-Titanium coil spring (0.0508×0.2032 mm, length=12 mm) as depicted in Figure 1. 

The springs were then fixed anteriorly using ligature-wire loops placed around the cervical third of the maxillary incisors. Spring activation generated 60 g of force when pulled from the first molar towards the upper incisors. The magnitude of orthodontic force was measured by a tension gauge. Upper incisors were considered as anchorage units during protraction of the first molars. During the experimental period, the rats were fed with powdered food and distilled water ad libitum. 

After 21 days of treatment, all rats were sacrificed with inhalant anesthesia (Halothane) and their maxilla was then removed. The amount of tooth movement (the space between the first and second molars) was measured using a Feeler Gauge (with an accuracy of 0.05 mm). 

## Reverse transcriptase-polymerase chain reaction study

The first molars were separated by using dissecting microscope and high speed fine diamond discs and burs. These teeth were examined under a stereomicroscope. The resorptive lacunae on the mesial surfaces of the subjects in the experimental group and the whole mesial root surface of control subjects were scratched with a fine diamond bur. The obtained tissues were frozen in liquid nitrogen. The frozen tissues were first crushed in a mortar and pestle. The resulting suspensions were then evaporated into disposable petri dishes, and 1 ml/g of tissue of RNX PLUS buffer (Sinaclon, Iran) was added to the suspension and incubated for 5 minutes at room temperature. An ultrasonic homogenizer device was used for further tissue homogenization. Next, 600 μL/g of tissue chloroform (The Science Company, USA) was added to the suspension and then transferred to centrifuge tubes, shaken well for 15 seconds and sustained at room temperature for a further 3 minutes. The samples were then centrifuged at 12000 xg for 15 minutes at 4˚C. After transferring the upper phase to new Eppendorf microtubes, 1.5 mg/l tissue isopropyl alcohol (Sciencelab, USA) was added. The samples were then incubated for 10 minutes, washed with 70% ethanol and centrifuged for 2 minutes at 12000 xg.

The precipitated RNA was then centrifuged at 12000 xg for 5 minutes at 4˚C and the RNA pellet was put on ice and air dried for 5 minutes at room temperature. Using the reverse transcriptase enzyme (QuantiTect, Germany) and 2 pmol of hexamer primers (Bioline, USA), cDNA was synthesized (at 42˚C for 1 hour) and then amplified using Taq DNA Polymerase, PCR buffer, specific primers (Roche, Germany) for TGF-β1 and glyceraldehyde 3-phosphate dehydrogenase gene (*GAPDH*, internal control/housekeeping gene), and MgCl_2_ under the following cycling conditions: 30 cycles of 30 seconds for denaturation at 94˚C, 30 seconds for annealing at 55˚C and 30 seconds for extension at 72˚C. The conventional RTPCR was performed using gene-specific primers. For loading control, identical amplification conditions were done with the housekeeping gene, *GAPDH*. The PCR products were then resolved by electrophoresis on a 2% w/v agarose gel and stained with ethidium bromide. PCR fragments were visualized using a UV trans illuminator. Based on RT-PCR of samples (case and control), normalized with *GAPDH* as an internal control (a housekeeping gene), the level of differential expression was estimated.

## Histologic evaluation

For histological evaluation, the maxillae of the second group of rats were first fixed in 10% neutral buffered formalin (Sigma, USA). The first molars and the surrounding bone in both test and control groups were block-dissected from the maxilla and decalcified in a 10% solution of ethylene diamine tetra-acetic acid (EDTA, Sigma, USA) until the specimens became soft. After complete decalcification, the specimens were rinsed in 0.01 M phosphate-buffered saline (PBS, Sigma, USA, pH=7.4) and dehydrated with increasing gradients of alcohol and methyl salicylate (Sciencelab, USA). Next, the tissues were embedded in paraffin with a melting point of 58-60˚C. Sagittal serial sections of 5 μm thickness were obtained and stained with hematoxylin and eosin (H&E).

The specimens were examined under a light microscope and the resorbed areas were examined with histomorphometric analysis. In the analysis, the external points of the resorptive areas were considered as reference points, and the area inscribed between the line joining the reference points and the resorbed surface was measured.

## Statistic analysis 

Data were represented as mean ± SD. To compare mean root resorption area in the test and control groups, t test was used. Mann Whitney U test was used to identify statistically significant differences in expression levels. SPSS (version 18) was used to implement the statistical test. The significance level was set at P<0.05. 

## Results

### Characterization of C2C12 differentiation

**Fig.2:**
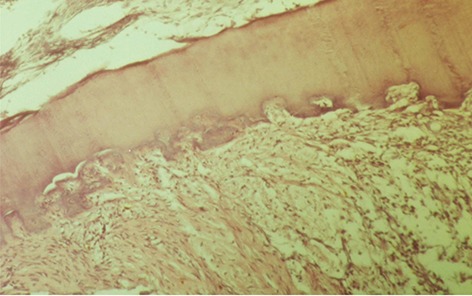
Photomicrograph of resorptive lacunae on mesial root surfaces along with multinucleated giant cell.

The increasing body weight of the samples showed that the intraoral appliances did not affect their nourishment. The average weight gain compared to the initial weight was 20%. The histological sections of the teeth subjected to orthodontic force revealed significant resorptive lacunae on mesial root surfaces with multinucleated giant cells present in these regions (Fig .2). None of these patterns were detected in the histological sections of control roots. This observation confirmed that the model used in this study was able to induce orthodontic tooth movement and root resorption. Based on histomorphometric analysis, the mean root resorption area in the test group was 4.8830×10^-11^ ± 0.163×10^-11^ mm^2^ while this was significantly lower in the control group at 1.7637×10^-11^ ± 0.503×10^-11^ mm^2^ (P<0.001). 

Comparison of TGF-β1 expression levels in test and control groups did not indicate any significant differential expression. 

## Discussion

Root resorption is an iatrogenic sequel of orthodontic treatment (1,10). More specifically, this is a biomechanical phenomenon because there is a relationship between OIIRR and biological inflammatory factors (11,12). Thus, in root resorption, it is essential to identify the role of biological inflammatory factors in decreasing the prevalence and severity of this phenomenon. Gene expression can be analyzed for diagnosis of early stages of different diseases like multiple sclerosis (13). However, lack of differential expression of TGF-β1 in root resorption makes this gene an unlikely biomarker candidate. 

Several researchers have evaluated the effect of tooth movement on root resorption. They showed that there is a direct relationship between these two variables (14,16), which is consistent with the findings of this study. Other studies have also shown that the role of *TGF-β1* in osteoblast and osteoclast differentiation is conflicting (8,17,20). Quinn et al. (21) studied the effect of *TGF-β1* on osteoclast activity and suggested that *TGF-β1* may increase osteoclast formation via action on osteoclast precursors. Nagia et al. (22) conducted a study to evaluate the role of *TGF-β1* in tooth movement. They concluded that *TGF-β1* is induced during tooth movement and that this inflammatory factor may regulate the adaptive alveolar bone modeling. Both these studies examined the relationship between *TGF-β1* expression and bone remodeling. Therefore, given that *TGF-β1* expression was not associated with root resorption, this association may be unique to the bone tissue. Lack of *TGF-β1* expression could thus be among the varieties that exist between these two hard tissue structures. 

## Conclusion

Based on the finding of this study, we confirm previous findings that there is a direct relationship between orthodontic force and orthodontic induced inflammatory root resorption. However, we observed no association between root resorption and inflammatory cytokine *TGF-β1* expression in the resorptive lacunae. 
